# Crystal structure of (+)-(1*S*,5*S*,6*S*,7*S*,10*S*,11*S*,16*S*)-16-hy­droxy-7-(meth­oxy­meth­oxy)-11,15,18,18-tetra­methyl-3,13-dioxo-2,4-dioxa­tetra­cyclo[12.3.1.0^1,5^.0^6,11^]octa­dec-14-en-10-yl benzoate

**DOI:** 10.1107/S2056989021011518

**Published:** 2021-11-04

**Authors:** Takeshi Oishi, Keisuke Fukaya, Takaaki Sato, Noritaka Chida

**Affiliations:** a School of Medicine, Keio University, Hiyoshi 4-1-1, Kohoku-ku, Yokohama 223-8521, Japan; b Department of Applied Chemistry, Faculty of Science and Technology, Keio University, Hiyoshi 3-14-1, Kohoku-ku, Yokohama 223-8522, Japan

**Keywords:** crystal structure, paclitaxel, taxane skeleton, dioxolane, cyclo­hexa­ne, cyclo­hexene, cyclo­octa­ne, hydrogen bond

## Abstract

In the fused tetra­cyclic system of the title compound, C_29_H_36_O_9_, the five-membered dioxolane ring adopts a twist conformation; the two adjacent C atoms deviate alternately from the mean plane of the other three atoms by −0.252 (6) and 0.340 (6) Å. The cyclo­hexane, cyclo­hexene and central cyclo­octane rings show chair, half-chair and boat-chair forms, respectively. There are three intra­molecular C—H⋯O inter­actions supporting the mol­ecular conformation, with one *S*(6) and two *S*(7) graph-set motifs. In the crystal, inter­molecular O—H⋯O hydrogen bonds connect the mol­ecules into a helical chain running along the *c*-axis direction, generating a *C*(7) graph-set motif. The chains are further linked by inter­molecular C—H⋯O inter­actions to construct a three-dimensional network. There is no valid C—H⋯π inter­action.

## Abstract 2

In the title compound, ring conformations in the tetra­cyclic system are twist, chair, half-chair and boat-chair forms. In the crystal, the inter­molecular O—H⋯O hydrogen bonds connect the mol­ecules into a helical chain, and the inter­molecular C—H⋯O inter­actions link the chains into a three-dimensional architecture.

## Chemical context

**Figure 1:**
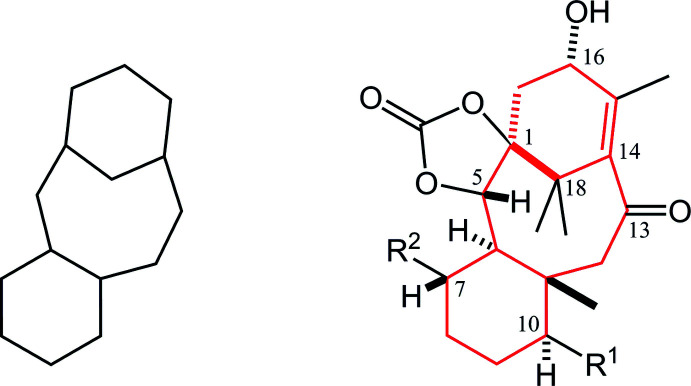
Left: Structure of tri­cyclo­[9.3.1.0^3,8^]penta­decane (taxane) skeleton. Right: Core structure of the title compound. Red lines indicate the taxane skeleton. *R* ^1^ = OC(=O)Ph, *R* ^2^ = OCH_2_OCH_3_.

Paclitaxel (systematic name: (1*S*,2*S*,3*R*,4*S*,7*R*,9*S*,10*S*,12*R*,15*S*)-4,12-diacet­oxy-1,9-dihy­droxy-15-{[(2*R*,3*S*)-3-benzoyl­amino-2-hy­droxy-3-phen­yl]propano­yl}­oxy-10,14,17,17-tetra­methyl-11-oxo-6-oxa-tetra­cyclo­[11.3.1.0^3,10^.0^4,7^]hepta­dec-13-en-2-yl benzoate) is a well-known natural diterpenoid con­taining a taxane framework (tri­cyclo­[9.3.1.0^3,8^]penta­decane; Fig. 1 ▸), with potent anti­tumor activity (Wall & Wani, 1995 ▸). Its highly complicated structure and significant bioactivity have attracted wide chemical and medicinal inter­est. 
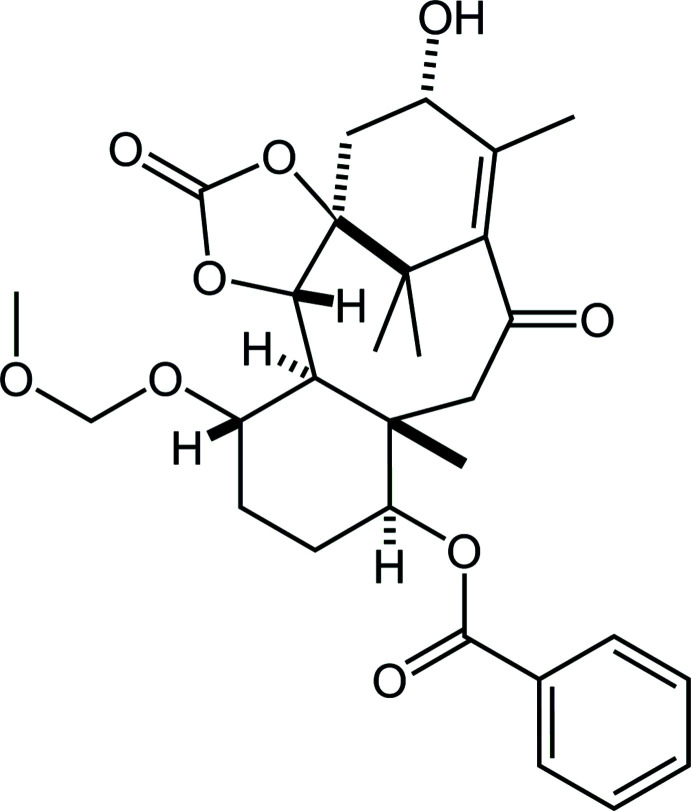


The title compound, which has a fused tetra­cyclic core composed of a taxane skeleton with an external cyclic carbonate, was afforded as a chiral form in an improved synthesis of paclitaxel (Iiyama *et al.*, 2021 ▸). Several closely related structures (Oishi, Yamaguchi *et al.*, 2015 ▸; Oishi, Fukaya *et al.*, 2015*a* ▸,*b* ▸) obtained in another synthetic pathway (Fukaya, Tanaka *et al.*, 2015 ▸; Fukaya, Kodama *et al.*, 2015 ▸) have been reported previously as racemic crystals.

## Structural commentary

**Figure 2:**
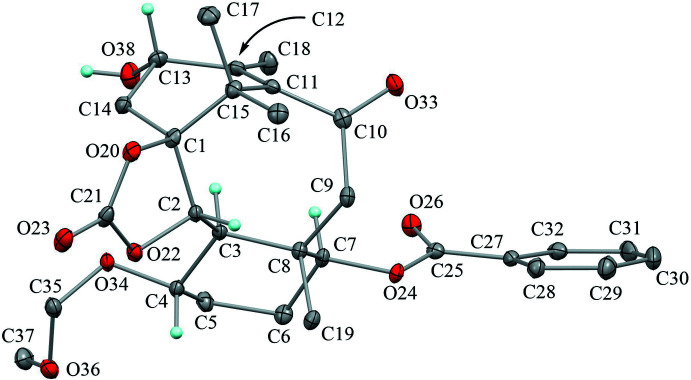
The mol­ecular structure of the title compound with the atom labels. Displacement ellipsoids are drawn at the 30% probability levels. Only H atoms connected to O and chiral C atoms are shown for clarity.

The mol­ecular structure of the title compound is shown in Fig. 2 ▸. The dioxolane ring (C1/C2/O22/C21/O20) adopts a twisted form with puckering parameters of *Q*(2) = 0.351 (2) Å and *φ*(2) = 56.6 (4)°. Atoms C1 and C2 deviate from the mean plane of the other three atoms by −0.250 (6) and 0.342 (6) Å, respectively. The cyclo­hexane ring (C3–C8) adopts a chair form with puckering parameters of *Q* = 0.580 (2) Å, *θ* = 8.0 (2)°, *φ* = 296.5 (17)°, *Q*(2) = 0.083 (2) Å and *Q*(3) = 0.574 (2) Å. The large substituents at C3, C4, C7 and C8 are in equatorial positions. The cyclo­hexene ring (C1/C14/C13/C12/C11/C15) adopts a half-chair form with puckering parameters of *Q* = 0.657 (3) Å, *θ* = 108.2 (3)°, *φ* = 135.8 (2)°, *Q*(2) = 0.624 (3) Å and *Q*(3) = −0.205 (3) Å. Atoms C1 and C14 deviate by 1.123 (4) and 0.811 (4) Å respectively, from the mean plane of the other four atoms with a maximum deviation of 0.0314 (15) Å at C12. The tetra­substituted olefin (C10/C15/C11=C12/C13/C18) is skewed from an ideal planar structure owing to strain in the fused-ring system. The torsion angles C10—C11=C12—C18, C15—C11=C12—C13, C10—C11=C12—C13 and C15—C11=C12—C18 are −14.1 (4), −7.0 (3), 159.6 (2) and 179.3 (2)°, respectively, and the dihedral angle between the C10/C11/C15 and C18/C12/C13 planes is 19.70 (17)°. The central cyclo­octane ring (C1–C3/C8–C11/C15) adopts a boat-chair form with puckering parameters of *Q* = 1.200 (2) Å, *Q*(2) = 0.948 (2) Å, *φ*(2) = 183.33 (15)°, *Q*(3) = 0.588 (2) Å, *φ*(3) = 3.3 (2)° and *Q*(4) = 0.444 (2) Å.

**Figure 3:**
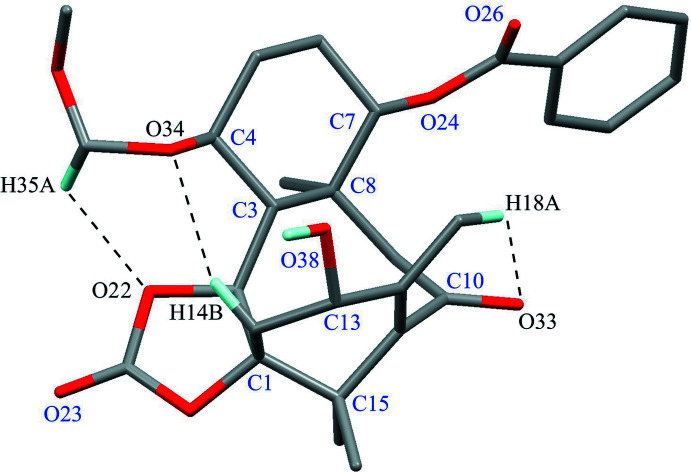
The mol­ecular conformation with the intra­molecular C—H⋯O inter­actions (black dashed lines). Only H atoms involved in these inter­actions and the hy­droxy H atom are shown for clarity.

There are three intra­molecular C—H⋯O inter­actions (C35—H35*A*⋯O22, C18—H18*A*⋯O33 and C14—H14*B*⋯O34; Table 1 ▸), generating *S*(7), *S*(6) and *S*(7) graph-set motifs, respectively (Fig. 3 ▸). The absolute structure was confirmed by the Flack parameter of 0.01 (7) with 1649 quotients [(*I* ^+^) − (*I* ^−^)]/[(*I* ^+^) + (*I* ^−^)] (Parsons *et al.*, 2013 ▸).

## Supra­molecular features

**Figure 4:**
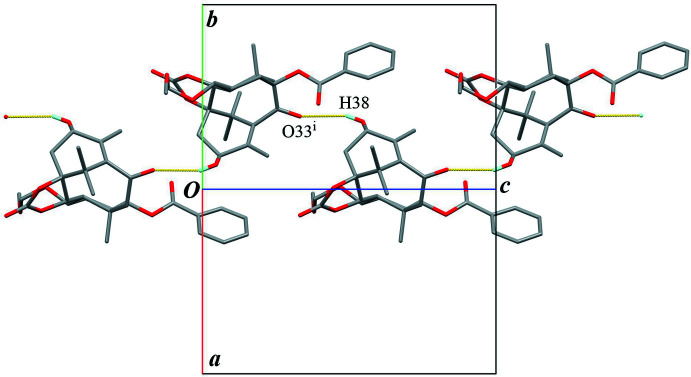
A partial packing diagram viewed down [110]. Yellow lines indicate the inter­molecular O—H⋯O hydrogen bonds. Only H atoms involved in the hydrogen bonds are shown for clarity. [Symmetry code: (i) −*x* + 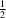 , −*y* + 1, *z* − 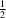 .]

**Figure 5:**
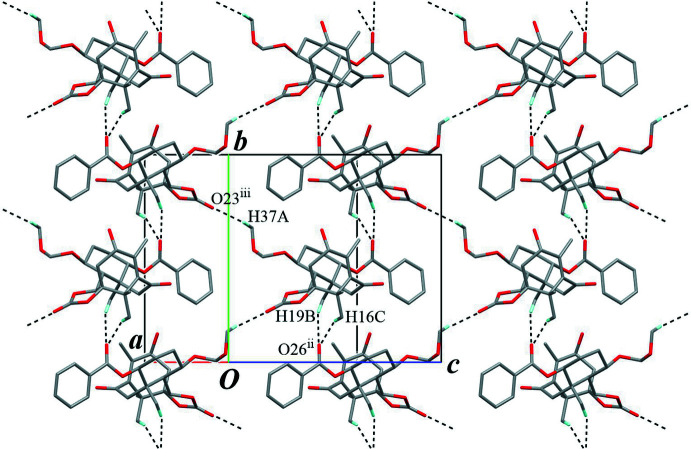
A partial packing diagram, showing the inter­molecular C—H⋯O inter­actions (black dashed lines) making a layer structure parallel to the (100) plane. Only H atoms involved in these inter­actions are shown for clarity. [Symmetry codes: (ii) −*x* + 1, *y* − 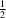 , −*z* + 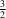 ; (iii) −*x* + 1, *y* + 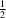 , −*z* + 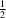 .]

The crystal packing is stabilized by an O—H⋯O hydrogen bond (O38—H38⋯O33^i^; symmetry code as given in Table 1 ▸), connecting the mol­ecules into a helical chain running along the *c-*axis direction, with a *C*(7) graph-set motif (Fig. 4 ▸). The chains are linked by an inter­molecular C—H⋯O hydrogen bond (C16—H16*C*⋯O26^ii^; Table 1 ▸) to build a three-dimensional architecture. Furthermore, two weak C—H⋯O inter­actions (C37—H37*A*⋯O23^iii^ and C19—H19*B*⋯O26^ii^; Table 1 ▸) support to form the network densely (Figs. 5 ▸ and 6 ▸). There is no valid C—H⋯π inter­action.

## Database survey

**Figure 7:**
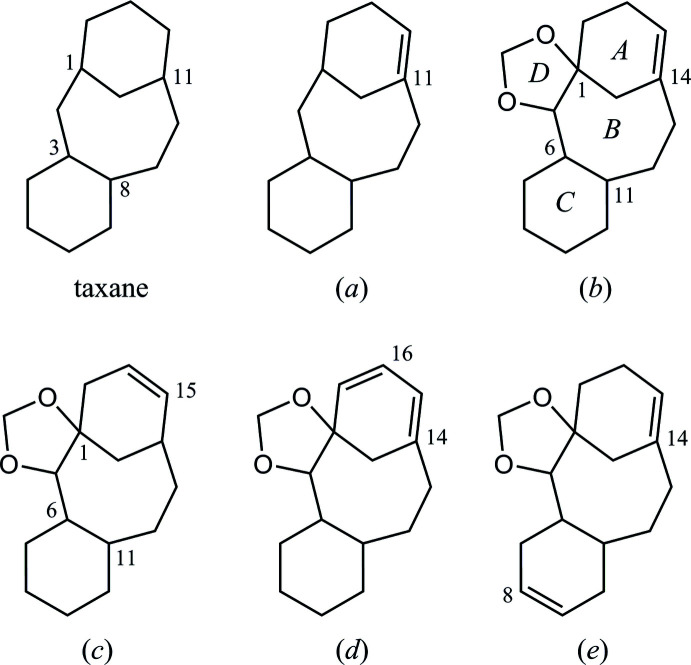
Core structures for database survey; tri­cyclo[9.3.1.0^3,8^]penta­decane (taxane) and its (*a*) 11-ene derivative, (*b*) 2,4-dioxa­tetra­cyclo[12.3.1.0^1,5^.0^6,11^]octa­dec-14-ene as the main frame of the title compound with ring-labelling, and its (*c*) regioisomer of olefin, (*d*) 16,17-de­hydro or (*e*) 8,9-de­hydro derivatives. The geometries of ring-fusion are similar to the title compound in every related structures, as *syn-AB*, *anti-BC* and *anti-BD*.

In the Cambridge Structural Database (CSD Version 5.42, last update September 2021; Groom *et al.*, 2016 ▸), 113 structures containing a tri­cyclo­[9.3.1.0^3,8^]penta­dec-11-ene skeleton, (*a*), are registered (Fig. 7 ▸). These include two chiral compounds (CSD refcodes NEGBOQ; Poujol *et al.*, 1997 ▸ and SUBQAJ; Hirai *et al.*, 2015 ▸) possessing a 2,4-dioxa­tetra­cyclo­[12.3.1.0^1,5^.0^6,11^]octa­dec-14-ene skeleton, (*b*), composed of *syn*-*AB*, *anti*-*BC* and *anti*-*BD* fused-ring systems similar to the title compound. Their ring conformations of the fused tetra­cycles (dioxolane, cyclo­hexane, cyclo­hexene and central cyclo­octa­ne) in the former structure are envelope, chair, half-chair and boat-chair forms, respectively, while those in the latter one are similar to the title compound as twist, chair, half-chair and boat-chair, respectively.

Four racemic structures closely related to the title compound, afforded by our previous synthesis, were also documented (XULNAV, XULMOI and XULMUO; Oishi, Fukaya *et al.*, 2015*a* ▸ and GUHWUD; Oishi, Fukaya *et al.*, 2015*b* ▸). For the former three structures, possessing a 2,4-dioxa­tetra­cyclo­[12.3.1.0^1,5^.0^6,11^]octa­dec-15-ene core, (*c*), their ring conformations of the tetra­cycles (dioxolane, cyclo­hexane, cyclo­hexene and central cyclo­octa­ne) are similar to one another as essentially planar, chair, half-chair and chair-chair forms, respectively. For the latter structure with a 2,4-dioxa­tetra­cyclo­[12.3.1.0^1,5^.0^6,11^]octa­deca-14,16-diene skeleton, (*d*), the ring conformations of dioxolane, cyclo­hexane, cyclo­hexene and central cyclo­octane are twist, chair, half-boat and boat-chair forms, respectively. Although two crystalline compounds with a 2,4-dioxa­tetra­cyclo­[12.3.1.0^1,5^.0^6,11^]octa­deca-8,14-diene skeleton, (*e*), have been reported (Nicolaou, Ueno *et al.*, 1995 ▸; Nicolaou, Yang *et al.*, 1995 ▸), they are not registered in the CSD.

## Synthesis and crystallization

The title compound was provided in an improved chiral synthesis of paclitaxel (Iiyama *et al.*, 2021 ▸). The cyclo­hexene unit (C1/C14/C13/C12/C11/C15) was prepared according to the reported procedure (Nicolaou, Liu *et al.*, 1995 ▸) from cyclo­hexa­ne-1,3-dione, while the tetra­substituted chiral cyclo­hexane unit (C3–C8) was derived from 3-meth­oxy­toluene (Fukaya *et al.*, 2016 ▸). Coupling reaction of these two units by utilizing a Shapiro reaction (Nicolaou, Liu *et al.*, 1995 ▸) led to generate the taxane framework, and further manipulations of the functional groups afforded the title compound. Purification was carried out by silica gel chromatography, and colorless crystals were obtained from a benzene solution under pentane-saturated atmosphere, by slow evaporation at ambient temperature. M.p. 505–508 K. [*α*]^27^_D_ + 13.2 (*c* 0.99, CHCl_3_). HRMS (ESI) *m/z* calculated for C_29_H_36_O_9_Na^+^ [*M* + Na]^+^: 551.2257; found: 551.2249.

## Refinement

Crystal data, data collection and structure refinement details are summarized in Table 2 ▸. C-bound H atoms were positioned geometrically with C—H = 0.95–1.00 Å, and constrained to ride on their parent atoms with *U*_iso_(H) = 1.2*U*_eq_(C) or 1.5*U*_eq_(methyl C). The hy­droxy H atom was located in a difference map and was allowed to refine as riding, with O—H = 0.84 Å, and with *U*_iso_(H) = 1.5*U*_eq_(O).

## Supplementary Material

Crystal structure: contains datablock(s) global, I. DOI: 10.1107/S2056989021011518/is5560sup1.cif

Structure factors: contains datablock(s) I. DOI: 10.1107/S2056989021011518/is5560Isup2.hkl

Supporting information file. DOI: 10.1107/S2056989021011518/is5560Isup3.cml

CCDC reference: 2119373

Additional supporting information:  crystallographic information; 3D view; checkCIF report

## Figures and Tables

**Figure 6:**
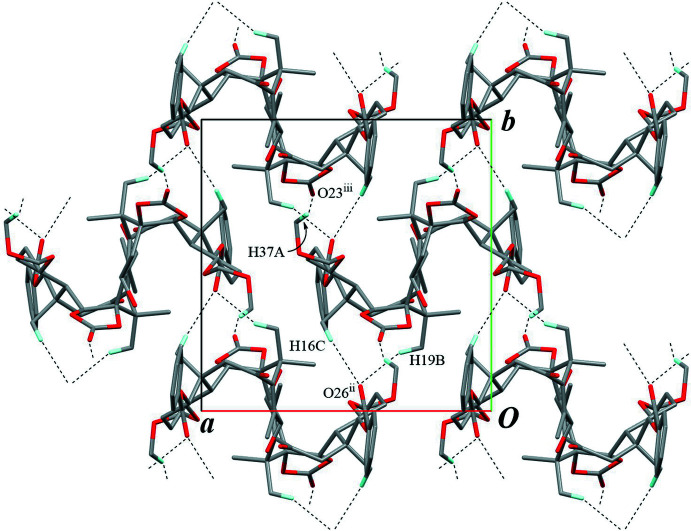
A packing diagram viewed down the *c* axis. Overlapping mol­ecules (projected as ‘N′ and inverted ‘N′ letter shapes) indicate the helical chains running along the *c* axis, which are connected by the inter­molecular C—H⋯O inter­actions (black dashed lines). Only H atoms involved in these inter­actions are shown for clarity. [Symmetry codes: (ii) −*x* + 1, *y* − 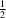 , −*z* + 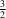 ; (iii) −*x* + 1, *y* + 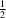 , −*z* + 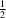 .]

**Table 1:** Hydrogen-bond geometry (Å, °)

*D*—H⋯*A*	*D*—H	H⋯*A*	*D*⋯*A*	*D*—H⋯*A*
O38—H38⋯O33^i^	0.84	2.49	3.251 (2)	151
C14—H14*B*⋯O34	0.99	2.57	3.423 (3)	145
C18—H18*A*⋯O33	0.98	2.53	3.244 (3)	130
C35—H35*A*⋯O22	0.99	2.36	2.990 (3)	121
C16—H16*C*⋯O26^ii^	0.98	2.43	3.331 (3)	153
C19—H19*B*⋯O26^ii^	0.98	2.59	3.534 (3)	162
C37—H37*A*⋯O23^iii^	0.98	2.52	3.445 (3)	158

Symmetry codes: (i) -x+{\script{1\over 2}}, -y+1, z-{\script{1\over 2}}; (ii) -x+1, y-{\script{1\over 2}}, -z+{\script{3\over 2}}; (iii) -x+1, y+{\script{1\over 2}}, -z+{\script{1\over 2}}.

**Table 2:** Experimental details

Crystal data	
Chemical formula	C_29_H_36_O_9_
*M*_r_	528.58
Crystal system, space group	Orthorhombic, *P*2_1_2_1_2_1_
Temperature (K)	90
*a*, *b*, *c* (Å)	13.2073 (2), 13.2580 (2), 14.8563 (2)
*V* (Å^3^)	2601.37 (7)
*Z*	4
Radiation type	Cu *K*α
μ (mm^−1^)	0.83
Crystal size (mm)	0.27 × 0.14 × 0.09

Data collection	
Diffractometer	Bruker D8 Venture
Absorption correction	Multi-scan (*SADABS*; Bruker, 2016 ▸)
*T*_min_, *T*_max_	0.84, 0.93
No. of measured, independent and observed [*I* > 2σ(*I*)] reflections	17552, 4488, 4049
*R*_int_	0.041
(sin θ/λ)_max_ (Å^−1^)	0.595

Refinement	
*R*[*F* ^2^ > 2σ(*F* ^2^)], *wR*(*F* ^2^), *S*	0.028, 0.058, 1.01
No. of reflections	4488
No. of parameters	349
H-atom treatment	H-atom parameters constrained
Δρ_max_, Δρ_min_ (e Å^−3^)	0.19, −0.17
Absolute structure	Flack *x* determined using 1649 quotients [(*I* ^+^)−(*I* ^−^)]/[(*I* ^+^)+(*I* ^−^)] (Parsons *et al.*, 2013 ▸).
Absolute structure parameter	0.01 (7)

Computer programs: *APEX3* and *SAINT* (Bruker, 2016 ▸), *SHELXT2014* (Sheldrick, 2015*a* ▸), *SHELXL2014* (Sheldrick, 2015*b* ▸), *Mercury* (Macrae *et al.*, 2020 ▸), *publCIF* (Westrip, 2010 ▸) and *PLATON* (Spek, 2020 ▸).

## supplementary crystallographic information

### Crystal data

**Table:**

C_29_H_36_O_9_	*D*_x_ = 1.350 Mg m^−^^3^
*M**_r_* = 528.58	Melting point = 508–505 K
Orthorhombic, *P*2_1_2_1_2_1_	Cu *K*α radiation, λ = 1.54178 Å
*a* = 13.2073 (2) Å	Cell parameters from 6149 reflections
*b* = 13.2580 (2) Å	θ = 4.5–66.6°
*c* = 14.8563 (2) Å	µ = 0.83 mm^−^^1^
*V* = 2601.37 (7) Å^3^	*T* = 90 K
*Z* = 4	Needle, colorless
*F*(000) = 1128	0.27 × 0.14 × 0.09 mm

### Data collection

**Table:**

Bruker D8 Venture diffractometer	4488 independent reflections
Radiation source: fine-focus sealed tube	4049 reflections with *I* > 2σ(*I*)
Multilayered confocal mirror monochromator	*R*_int_ = 0.041
Detector resolution: 10.4167 pixels mm^-1^	θ_max_ = 66.6°, θ_min_ = 4.5°
φ and ω scans	*h* = −15→15
Absorption correction: multi-scan (*SADABS*; Bruker, 2016)	*k* = −13→15
*T*_min_ = 0.84, *T*_max_ = 0.93	*l* = −17→17
17552 measured reflections	

### Refinement

**Table:**

Refinement on *F*^2^	Secondary atom site location: difference Fourier map
Least-squares matrix: full	Hydrogen site location: inferred from neighbouring sites
*R*[*F*^2^ > 2σ(*F*^2^)] = 0.028	H-atom parameters constrained
*wR*(*F*^2^) = 0.058	*w* = 1/[σ^2^(*F*_o_^2^) + 0.9512*P*] where *P* = (*F*_o_^2^ + 2*F*_c_^2^)/3
*S* = 1.00	(Δ/σ)_max_ < 0.001
4488 reflections	Δρ_max_ = 0.19 e Å^−^^3^
349 parameters	Δρ_min_ = −0.17 e Å^−^^3^
0 restraints	Absolute structure: Flack *x* determined using 1649 quotients [(*I*^+^)-(*I*^-^)]/[(*I*^+^)+(*I*^-^)] (Parsons *et al.*, 2013).
Primary atom site location: structure-invariant direct methods	Absolute structure parameter: 0.01 (7)

### Special details

**Table:**

Geometry. All esds (except the esd in the dihedral angle between two l.s. planes) are estimated using the full covariance matrix. The cell esds are taken into account individually in the estimation of esds in distances, angles and torsion angles; correlations between esds in cell parameters are only used when they are defined by crystal symmetry. An approximate (isotropic) treatment of cell esds is used for estimating esds involving l.s. planes.
Refinement. Refinement of *F*^2^ against ALL reflections. The weighted *R*–factor *wR* and goodness of fit *S* are based on *F*^2^, conventional *R*–factors *R* are based on *F*, with *F* set to zero for negative *F*^2^. The threshold expression of *F*^2^ > 2*σ*(*F*^2^) is used only for calculating *R*–factors(gt) *etc.* and is not relevant to the choice of reflections for refinement. *R*–factors based on *F*^2^ are statistically about twice as large as those based on *F*, and *R*–factors based on ALL data will be even larger. Problematic ten reflections (1 7 0, 0 9 1, 5 1 5, 0 0 8, 1 11 1, 2 7 0, –2 13 2, 2 1 7, 1 12 2, 2 13 2) with |*I*(obs)-*I*(calc)|/*σ*W(*I*) greater than 10 have been omitted in the final refinement.

### Fractional atomic coordinates and isotropic or equivalent isotropic displacement parameters (Å^2^)

**Table:**

	*x*	*y*	*z*	*U*_iso_*/*U*_eq_	
C1	0.29313 (18)	0.36157 (17)	0.52288 (15)	0.0185 (5)	
C2	0.40702 (17)	0.34711 (17)	0.54120 (14)	0.0165 (5)	
H2	0.4148	0.2822	0.5748	0.02*	
C3	0.46542 (17)	0.42818 (16)	0.59392 (14)	0.0154 (5)	
H3	0.4159	0.4832	0.6073	0.019*	
C4	0.55414 (18)	0.47775 (16)	0.54327 (14)	0.0170 (5)	
H4	0.6045	0.4258	0.524	0.02*	
C5	0.60464 (18)	0.55792 (19)	0.60195 (15)	0.0222 (5)	
H5A	0.6669	0.5815	0.5713	0.027*	
H5B	0.5584	0.6164	0.6072	0.027*	
C6	0.63253 (18)	0.52209 (18)	0.69649 (15)	0.0199 (5)	
H6A	0.6888	0.4729	0.6928	0.024*	
H6B	0.6558	0.5802	0.7328	0.024*	
C7	0.54246 (17)	0.47339 (16)	0.74180 (14)	0.0171 (5)	
H7	0.4881	0.525	0.7503	0.02*	
C8	0.49959 (17)	0.38399 (17)	0.68729 (14)	0.0163 (5)	
C9	0.41449 (18)	0.33002 (18)	0.74281 (15)	0.0190 (5)	
H9A	0.405	0.2628	0.7151	0.023*	
H9B	0.4427	0.3181	0.8036	0.023*	
C10	0.30917 (18)	0.37388 (17)	0.75641 (16)	0.0192 (5)	
C11	0.25421 (17)	0.40784 (17)	0.67406 (15)	0.0184 (5)	
C12	0.24790 (18)	0.50668 (17)	0.65522 (16)	0.0194 (5)	
C13	0.22472 (18)	0.54081 (18)	0.55976 (15)	0.0219 (5)	
H13	0.1498	0.5487	0.5533	0.026*	
C14	0.26259 (19)	0.46571 (18)	0.48757 (15)	0.0206 (5)	
H14A	0.2085	0.4568	0.4421	0.025*	
H14B	0.3217	0.4959	0.4566	0.025*	
C15	0.22581 (18)	0.32944 (18)	0.60119 (15)	0.0203 (5)	
C16	0.23971 (19)	0.21823 (17)	0.62803 (16)	0.0240 (5)	
H16A	0.2024	0.1751	0.5859	0.036*	
H16B	0.2137	0.2078	0.6891	0.036*	
H16C	0.3118	0.2009	0.6262	0.036*	
C17	0.11267 (19)	0.3400 (2)	0.57840 (17)	0.0283 (6)	
H17A	0.0972	0.4108	0.5652	0.042*	
H17B	0.072	0.3175	0.6298	0.042*	
H17C	0.0967	0.2985	0.5257	0.042*	
C18	0.2724 (2)	0.58910 (19)	0.72090 (16)	0.0278 (6)	
H18A	0.2873	0.5595	0.7799	0.042*	
H18B	0.2143	0.6347	0.7262	0.042*	
H18C	0.3314	0.6269	0.6995	0.042*	
C19	0.58214 (18)	0.30291 (17)	0.67462 (16)	0.0197 (5)	
H19A	0.6384	0.3312	0.6398	0.03*	
H19B	0.5536	0.245	0.6424	0.03*	
H19C	0.6069	0.281	0.7336	0.03*	
O20	0.28228 (12)	0.29062 (12)	0.44657 (10)	0.0221 (4)	
C21	0.37150 (19)	0.28495 (18)	0.40342 (16)	0.0220 (5)	
O22	0.44552 (12)	0.33066 (11)	0.45053 (10)	0.0193 (4)	
O23	0.38417 (14)	0.24438 (14)	0.33279 (11)	0.0302 (4)	
O24	0.57161 (12)	0.43307 (11)	0.82891 (10)	0.0175 (3)	
C25	0.56613 (17)	0.49480 (17)	0.90059 (15)	0.0177 (5)	
O26	0.54871 (13)	0.58399 (12)	0.89538 (11)	0.0247 (4)	
C27	0.57910 (17)	0.43860 (18)	0.98644 (15)	0.0184 (5)	
C28	0.56861 (19)	0.33481 (19)	0.99105 (16)	0.0241 (6)	
H28	0.5596	0.2971	0.9373	0.029*	
C29	0.5710 (2)	0.2853 (2)	1.07304 (17)	0.0320 (6)	
H29	0.5636	0.2141	1.0758	0.038*	
C30	0.5845 (2)	0.3411 (2)	1.15120 (17)	0.0355 (7)	
H30	0.5836	0.3082	1.208	0.043*	
C31	0.5993 (2)	0.4441 (2)	1.14701 (16)	0.0326 (7)	
H31	0.6116	0.4812	1.2006	0.039*	
C32	0.59611 (17)	0.4935 (2)	1.06494 (16)	0.0237 (6)	
H32	0.6055	0.5645	1.0622	0.028*	
O33	0.27312 (13)	0.37839 (13)	0.83187 (11)	0.0279 (4)	
O34	0.51273 (12)	0.52911 (12)	0.46645 (10)	0.0189 (4)	
C35	0.56149 (19)	0.50924 (19)	0.38360 (15)	0.0235 (5)	
H35A	0.559	0.4358	0.3719	0.028*	
H35B	0.5235	0.5433	0.3348	0.028*	
O36	0.66209 (13)	0.54080 (13)	0.38042 (11)	0.0262 (4)	
C37	0.6726 (2)	0.6481 (2)	0.37659 (18)	0.0299 (6)	
H37A	0.6369	0.674	0.3236	0.045*	
H37B	0.7445	0.6657	0.3725	0.045*	
H37C	0.6436	0.6782	0.4311	0.045*	
O38	0.27143 (15)	0.63683 (12)	0.54668 (12)	0.0311 (4)	
H38	0.2602	0.6569	0.494	0.047*	

### Atomic displacement parameters (Å^2^)

**Table:**

	*U*^11^	*U*^22^	*U*^33^	*U*^12^	*U*^13^	*U*^23^
C1	0.0221 (13)	0.0182 (13)	0.0153 (12)	−0.0001 (9)	−0.0062 (10)	−0.0049 (9)
C2	0.0206 (12)	0.0169 (13)	0.0121 (11)	0.0014 (9)	0.0000 (9)	0.0006 (9)
C3	0.0181 (12)	0.0143 (12)	0.0139 (10)	0.0033 (9)	−0.0001 (10)	−0.0004 (9)
C4	0.0198 (12)	0.0166 (13)	0.0146 (11)	0.0021 (9)	−0.0008 (10)	0.0015 (9)
C5	0.0231 (13)	0.0230 (14)	0.0204 (12)	−0.0060 (10)	0.0013 (11)	0.0029 (10)
C6	0.0221 (13)	0.0172 (14)	0.0204 (12)	−0.0046 (9)	−0.0034 (10)	−0.0015 (9)
C7	0.0237 (13)	0.0151 (13)	0.0124 (11)	0.0021 (9)	−0.0030 (9)	0.0005 (9)
C8	0.0187 (12)	0.0156 (12)	0.0148 (11)	−0.0003 (9)	−0.0017 (10)	0.0007 (9)
C9	0.0251 (14)	0.0174 (12)	0.0144 (11)	−0.0032 (9)	−0.0033 (10)	0.0012 (9)
C10	0.0255 (13)	0.0129 (12)	0.0193 (13)	−0.0056 (9)	0.0010 (11)	−0.0023 (9)
C11	0.0141 (12)	0.0226 (14)	0.0184 (11)	0.0001 (9)	0.0031 (10)	−0.0020 (9)
C12	0.0162 (11)	0.0219 (13)	0.0202 (12)	0.0033 (9)	0.0029 (10)	−0.0032 (9)
C13	0.0218 (13)	0.0201 (13)	0.0238 (12)	0.0048 (10)	0.0001 (10)	0.0005 (10)
C14	0.0207 (13)	0.0241 (13)	0.0169 (11)	0.0034 (10)	−0.0029 (10)	−0.0004 (9)
C15	0.0196 (13)	0.0208 (13)	0.0204 (12)	−0.0012 (10)	−0.0026 (10)	−0.0003 (10)
C16	0.0246 (13)	0.0214 (13)	0.0258 (13)	−0.0052 (10)	−0.0020 (11)	−0.0016 (10)
C17	0.0220 (14)	0.0322 (16)	0.0308 (14)	−0.0043 (11)	−0.0020 (11)	−0.0027 (11)
C18	0.0402 (16)	0.0227 (14)	0.0206 (12)	0.0036 (11)	0.0015 (12)	−0.0039 (10)
C19	0.0241 (13)	0.0185 (13)	0.0166 (11)	0.0019 (9)	−0.0047 (10)	0.0001 (9)
O20	0.0252 (9)	0.0228 (9)	0.0182 (8)	0.0006 (7)	−0.0064 (7)	−0.0071 (7)
C21	0.0291 (14)	0.0180 (13)	0.0190 (12)	0.0069 (10)	−0.0067 (11)	−0.0015 (10)
O22	0.0238 (9)	0.0204 (9)	0.0138 (8)	0.0029 (7)	−0.0007 (7)	−0.0039 (6)
O23	0.0401 (11)	0.0314 (11)	0.0189 (9)	0.0117 (8)	−0.0075 (8)	−0.0098 (8)
O24	0.0239 (9)	0.0150 (9)	0.0134 (7)	0.0014 (6)	−0.0047 (7)	−0.0004 (6)
C25	0.0161 (11)	0.0186 (14)	0.0183 (11)	−0.0016 (9)	−0.0003 (10)	−0.0035 (9)
O26	0.0350 (10)	0.0170 (10)	0.0221 (9)	0.0007 (7)	0.0006 (8)	−0.0025 (7)
C27	0.0149 (12)	0.0245 (14)	0.0159 (11)	0.0008 (9)	−0.0006 (10)	0.0000 (9)
C28	0.0253 (14)	0.0276 (15)	0.0193 (12)	−0.0007 (11)	−0.0033 (11)	0.0005 (10)
C29	0.0337 (15)	0.0334 (16)	0.0287 (15)	−0.0021 (12)	−0.0025 (12)	0.0114 (11)
C30	0.0332 (16)	0.055 (2)	0.0186 (13)	0.0010 (13)	−0.0009 (12)	0.0112 (12)
C31	0.0275 (14)	0.055 (2)	0.0150 (12)	0.0018 (13)	−0.0016 (11)	−0.0082 (12)
C32	0.0188 (13)	0.0300 (15)	0.0224 (13)	0.0014 (10)	−0.0001 (10)	−0.0056 (10)
O33	0.0354 (10)	0.0296 (10)	0.0188 (9)	0.0012 (8)	0.0062 (8)	0.0013 (7)
O34	0.0247 (9)	0.0197 (9)	0.0124 (8)	0.0025 (6)	0.0011 (7)	0.0023 (6)
C35	0.0290 (14)	0.0253 (14)	0.0163 (12)	0.0019 (11)	0.0031 (10)	0.0004 (9)
O36	0.0272 (10)	0.0276 (10)	0.0236 (9)	0.0022 (7)	0.0067 (7)	0.0051 (7)
C37	0.0298 (15)	0.0291 (16)	0.0307 (14)	−0.0024 (11)	0.0034 (12)	0.0113 (11)
O38	0.0482 (12)	0.0182 (10)	0.0269 (9)	0.0012 (8)	0.0030 (9)	0.0029 (7)

### Geometric parameters (Å, º)

**Table:**

C1—O20	1.480 (3)	C15—C17	1.539 (3)
C1—C14	1.531 (3)	C16—H16A	0.98
C1—C15	1.525 (3)	C16—H16B	0.98
C1—C2	1.541 (3)	C16—H16C	0.98
C2—O22	1.456 (3)	C17—H17A	0.98
C2—C3	1.537 (3)	C17—H17B	0.98
C2—H2	1.0	C17—H17C	0.98
C3—C4	1.540 (3)	C18—H18A	0.98
C3—C8	1.572 (3)	C18—H18B	0.98
C3—H3	1.0	C18—H18C	0.98
C4—O34	1.437 (3)	C19—H19A	0.98
C4—C5	1.528 (3)	C19—H19B	0.98
C4—H4	1.0	C19—H19C	0.98
C5—C6	1.528 (3)	O20—C21	1.344 (3)
C5—H5A	0.99	C21—O23	1.191 (3)
C5—H5B	0.99	C21—O22	1.346 (3)
C6—C7	1.512 (3)	O24—C25	1.345 (3)
C6—H6A	0.99	C25—O26	1.207 (3)
C6—H6B	0.99	C25—C27	1.487 (3)
C7—O24	1.452 (3)	C27—C28	1.385 (4)
C7—C8	1.543 (3)	C27—C32	1.393 (3)
C7—H7	1.0	C28—C29	1.384 (3)
C8—C19	1.543 (3)	C28—H28	0.95
C8—C9	1.567 (3)	C29—C30	1.389 (4)
C9—C10	1.521 (3)	C29—H29	0.95
C9—H9A	0.99	C30—C31	1.380 (4)
C9—H9B	0.99	C30—H30	0.95
C10—O33	1.219 (3)	C31—C32	1.385 (4)
C10—C11	1.492 (3)	C31—H31	0.95
C11—C12	1.343 (3)	C32—H32	0.95
C11—C15	1.547 (3)	O34—C35	1.414 (3)
C12—C18	1.500 (3)	C35—O36	1.394 (3)
C12—C13	1.520 (3)	C35—H35A	0.99
C13—O38	1.428 (3)	C35—H35B	0.99
C13—C14	1.547 (3)	O36—C37	1.431 (3)
C13—H13	1.0	C37—H37A	0.98
C14—H14A	0.99	C37—H37B	0.98
C14—H14B	0.99	C37—H37C	0.98
C15—C16	1.538 (3)	O38—H38	0.84
			
O20—C1—C14	106.57 (17)	H14A—C14—H14B	107.5
O20—C1—C15	110.51 (18)	C16—C15—C1	113.3 (2)
C14—C1—C15	111.1 (2)	C16—C15—C11	115.70 (19)
O20—C1—C2	98.67 (17)	C1—C15—C11	101.82 (18)
C14—C1—C2	115.5 (2)	C16—C15—C17	105.1 (2)
C15—C1—C2	113.56 (18)	C1—C15—C17	111.90 (19)
O22—C2—C1	101.31 (16)	C11—C15—C17	109.2 (2)
O22—C2—C3	113.62 (18)	C15—C16—H16A	109.5
C1—C2—C3	119.53 (18)	C15—C16—H16B	109.5
O22—C2—H2	107.2	H16A—C16—H16B	109.5
C1—C2—H2	107.2	C15—C16—H16C	109.5
C3—C2—H2	107.2	H16A—C16—H16C	109.5
C4—C3—C2	115.54 (18)	H16B—C16—H16C	109.5
C4—C3—C8	111.83 (18)	C15—C17—H17A	109.5
C2—C3—C8	109.45 (17)	C15—C17—H17B	109.5
C4—C3—H3	106.5	H17A—C17—H17B	109.5
C2—C3—H3	106.5	C15—C17—H17C	109.5
C8—C3—H3	106.5	H17A—C17—H17C	109.5
O34—C4—C5	106.83 (17)	H17B—C17—H17C	109.5
O34—C4—C3	107.50 (18)	C12—C18—H18A	109.5
C5—C4—C3	110.50 (18)	C12—C18—H18B	109.5
O34—C4—H4	110.6	H18A—C18—H18B	109.5
C5—C4—H4	110.6	C12—C18—H18C	109.5
C3—C4—H4	110.6	H18A—C18—H18C	109.5
C6—C5—C4	114.42 (19)	H18B—C18—H18C	109.5
C6—C5—H5A	108.7	C8—C19—H19A	109.5
C4—C5—H5A	108.7	C8—C19—H19B	109.5
C6—C5—H5B	108.7	H19A—C19—H19B	109.5
C4—C5—H5B	108.7	C8—C19—H19C	109.5
H5A—C5—H5B	107.6	H19A—C19—H19C	109.5
C7—C6—C5	110.63 (19)	H19B—C19—H19C	109.5
C7—C6—H6A	109.5	C21—O20—C1	108.42 (17)
C5—C6—H6A	109.5	O23—C21—O22	124.0 (2)
C7—C6—H6B	109.5	O23—C21—O20	124.7 (2)
C5—C6—H6B	109.5	O22—C21—O20	111.32 (19)
H6A—C6—H6B	108.1	C21—O22—C2	107.14 (17)
O24—C7—C6	110.20 (18)	C25—O24—C7	117.86 (17)
O24—C7—C8	106.39 (17)	O26—C25—O24	123.7 (2)
C6—C7—C8	112.53 (18)	O26—C25—C27	124.6 (2)
O24—C7—H7	109.2	O24—C25—C27	111.59 (19)
C6—C7—H7	109.2	C28—C27—C32	119.6 (2)
C8—C7—H7	109.2	C28—C27—C25	121.9 (2)
C19—C8—C7	109.88 (18)	C32—C27—C25	118.3 (2)
C19—C8—C9	104.65 (18)	C27—C28—C29	120.8 (2)
C7—C8—C9	109.74 (17)	C27—C28—H28	119.6
C19—C8—C3	110.79 (17)	C29—C28—H28	119.6
C7—C8—C3	106.39 (17)	C28—C29—C30	119.1 (3)
C9—C8—C3	115.39 (18)	C28—C29—H29	120.5
C10—C9—C8	123.5 (2)	C30—C29—H29	120.5
C10—C9—H9A	106.5	C31—C30—C29	120.5 (2)
C8—C9—H9A	106.5	C31—C30—H30	119.7
C10—C9—H9B	106.5	C29—C30—H30	119.7
C8—C9—H9B	106.5	C30—C31—C32	120.2 (2)
H9A—C9—H9B	106.5	C30—C31—H31	119.9
O33—C10—C11	123.3 (2)	C32—C31—H31	119.9
O33—C10—C9	119.9 (2)	C31—C32—C27	119.7 (3)
C11—C10—C9	116.8 (2)	C31—C32—H32	120.2
C12—C11—C10	119.7 (2)	C27—C32—H32	120.2
C12—C11—C15	119.7 (2)	C35—O34—C4	115.45 (17)
C10—C11—C15	119.28 (19)	O36—C35—O34	114.06 (19)
C11—C12—C18	124.2 (2)	O36—C35—H35A	108.7
C11—C12—C13	119.8 (2)	O34—C35—H35A	108.7
C18—C12—C13	115.7 (2)	O36—C35—H35B	108.7
O38—C13—C12	107.78 (19)	O34—C35—H35B	108.7
O38—C13—C14	109.87 (19)	H35A—C35—H35B	107.6
C12—C13—C14	112.97 (19)	C35—O36—C37	113.11 (19)
O38—C13—H13	108.7	O36—C37—H37A	109.5
C12—C13—H13	108.7	O36—C37—H37B	109.5
C14—C13—H13	108.7	H37A—C37—H37B	109.5
C1—C14—C13	115.35 (19)	O36—C37—H37C	109.5
C1—C14—H14A	108.4	H37A—C37—H37C	109.5
C13—C14—H14A	108.4	H37B—C37—H37C	109.5
C1—C14—H14B	108.4	C13—O38—H38	109.5
C13—C14—H14B	108.4		
			
O20—C1—C2—O22	34.44 (19)	C15—C1—C14—C13	34.9 (3)
C14—C1—C2—O22	−78.6 (2)	C2—C1—C14—C13	−96.3 (2)
C15—C1—C2—O22	151.38 (18)	O38—C13—C14—C1	134.1 (2)
O20—C1—C2—C3	160.10 (18)	C12—C13—C14—C1	13.7 (3)
C14—C1—C2—C3	47.0 (3)	O20—C1—C15—C16	51.3 (2)
C15—C1—C2—C3	−83.0 (3)	C14—C1—C15—C16	169.37 (19)
O22—C2—C3—C4	−0.3 (3)	C2—C1—C15—C16	−58.5 (3)
C1—C2—C3—C4	−119.9 (2)	O20—C1—C15—C11	176.24 (17)
O22—C2—C3—C8	−127.57 (19)	C14—C1—C15—C11	−65.7 (2)
C1—C2—C3—C8	112.8 (2)	C2—C1—C15—C11	66.4 (2)
C2—C3—C4—O34	62.8 (2)	O20—C1—C15—C17	−67.3 (3)
C8—C3—C4—O34	−171.15 (16)	C14—C1—C15—C17	50.8 (3)
C2—C3—C4—C5	179.00 (18)	C2—C1—C15—C17	−177.11 (19)
C8—C3—C4—C5	−54.9 (2)	C12—C11—C15—C16	177.6 (2)
O34—C4—C5—C6	167.08 (19)	C10—C11—C15—C16	10.9 (3)
C3—C4—C5—C6	50.4 (3)	C12—C11—C15—C1	54.3 (3)
C4—C5—C6—C7	−51.3 (3)	C10—C11—C15—C1	−112.5 (2)
C5—C6—C7—O24	175.86 (18)	C12—C11—C15—C17	−64.2 (3)
C5—C6—C7—C8	57.3 (3)	C10—C11—C15—C17	129.1 (2)
O24—C7—C8—C19	−61.5 (2)	C14—C1—O20—C21	91.8 (2)
C6—C7—C8—C19	59.3 (2)	C15—C1—O20—C21	−147.45 (19)
O24—C7—C8—C9	53.1 (2)	C2—C1—O20—C21	−28.2 (2)
C6—C7—C8—C9	173.87 (19)	C1—O20—C21—O23	−170.0 (2)
O24—C7—C8—C3	178.56 (17)	C1—O20—C21—O22	10.3 (2)
C6—C7—C8—C3	−60.7 (2)	O23—C21—O22—C2	−165.5 (2)
C4—C3—C8—C19	−60.1 (2)	O20—C21—O22—C2	14.2 (2)
C2—C3—C8—C19	69.2 (2)	C1—C2—O22—C21	−31.1 (2)
C4—C3—C8—C7	59.2 (2)	C3—C2—O22—C21	−160.57 (18)
C2—C3—C8—C7	−171.41 (18)	C6—C7—O24—C25	88.2 (2)
C4—C3—C8—C9	−178.81 (18)	C8—C7—O24—C25	−149.56 (18)
C2—C3—C8—C9	−49.5 (2)	C7—O24—C25—O26	−8.0 (3)
C19—C8—C9—C10	−168.0 (2)	C7—O24—C25—C27	169.16 (18)
C7—C8—C9—C10	74.2 (3)	O26—C25—C27—C28	160.2 (2)
C3—C8—C9—C10	−45.9 (3)	O24—C25—C27—C28	−17.0 (3)
C8—C9—C10—O33	−130.2 (2)	O26—C25—C27—C32	−15.8 (4)
C8—C9—C10—C11	50.9 (3)	O24—C25—C27—C32	167.0 (2)
O33—C10—C11—C12	77.9 (3)	C32—C27—C28—C29	2.4 (4)
C9—C10—C11—C12	−103.3 (3)	C25—C27—C28—C29	−173.5 (2)
O33—C10—C11—C15	−115.4 (3)	C27—C28—C29—C30	−0.2 (4)
C9—C10—C11—C15	63.4 (3)	C28—C29—C30—C31	−2.5 (4)
C10—C11—C12—C18	−14.1 (4)	C29—C30—C31—C32	3.0 (4)
C15—C11—C12—C18	179.3 (2)	C30—C31—C32—C27	−0.7 (4)
C10—C11—C12—C13	159.6 (2)	C28—C27—C32—C31	−2.0 (4)
C15—C11—C12—C13	−7.0 (3)	C25—C27—C32—C31	174.1 (2)
C11—C12—C13—O38	−150.6 (2)	C5—C4—O34—C35	110.5 (2)
C18—C12—C13—O38	23.6 (3)	C3—C4—O34—C35	−130.85 (19)
C11—C12—C13—C14	−29.0 (3)	C4—O34—C35—O36	−63.4 (3)
C18—C12—C13—C14	145.2 (2)	O34—C35—O36—C37	−71.5 (3)
O20—C1—C14—C13	155.30 (19)		

### Hydrogen-bond geometry (Å, º)

**Table:**

*D*—H···*A*	*D*—H	H···*A*	*D*···*A*	*D*—H···*A*
O38—H38···O33^i^	0.84	2.49	3.251 (2)	151
C14—H14*B*···O34	0.99	2.57	3.423 (3)	145
C18—H18*A*···O33	0.98	2.53	3.244 (3)	130
C35—H35*A*···O22	0.99	2.36	2.990 (3)	121
C16—H16*C*···O26^ii^	0.98	2.43	3.331 (3)	153
C19—H19*B*···O26^ii^	0.98	2.59	3.534 (3)	162
C37—H37*A*···O23^iii^	0.98	2.52	3.445 (3)	158

Symmetry codes: (i) −*x*+1/2, −*y*+1, *z*−1/2; (ii) −*x*+1, *y*−1/2, −*z*+3/2; (iii) −*x*+1, *y*+1/2, −*z*+1/2.
